# Factors Influencing Obstacle Crossing Performance in Patients with Parkinson's Disease

**DOI:** 10.1371/journal.pone.0084245

**Published:** 2014-01-13

**Authors:** Ying-Yi Liao, Yea-Ru Yang, Yih-Ru Wu, Ray-Yau Wang

**Affiliations:** 1 Department of Physical Therapy and Assistive Technology, National Yang-Ming University, Taipei, Taiwan; 2 Department of Rehabilitation, Jen-Teh Junior College of Medicine, Nursing and Management, Miaoli, Taiwan; 3 Department of Neuromuscular Disorders, Chang Guan Memorial Hospital, Linkou, Taiwan; Hospital General Dr. Manuel Gea González, Mexico

## Abstract

**Background:**

Tripping over obstacles is the major cause of falls in community-dwelling patients with Parkinson's disease (PD). Understanding the factors associated with the obstacle crossing behavior may help to develop possible training programs for crossing performance. This study aimed to identify the relationships and important factors determining obstacle crossing performance in patients with PD.

**Methods:**

Forty-two idiopathic patients with PD (Hoehn and Yahr stages I to III) participated in this study. Obstacle crossing performance was recorded by the Liberty system, a three-dimensional motion capture device. Maximal isometric strength of the lower extremity was measured by a handheld dynamometer. Dynamic balance and sensory integration ability were assessed using the Balance Master system. Movement velocity (MV), maximal excursion (ME), and directional control (DC) were obtained during the limits of stability test to quantify dynamic balance. The sum of sensory organization test (SOT) scores was used to quantify sensory organization ability.

**Results:**

Both crossing stride length and stride velocity correlated significantly with lower extremity muscle strength, dynamic balance control (forward and sideward), and sum of SOT scores. From the regression model, forward DC and ankle dorsiflexor strength were identified as two major determinants for crossing performance (R^2^ = .37 to.41 for the crossing stride length, R^2^ = .43 to.44 for the crossing stride velocity).

**Conclusions:**

Lower extremity muscle strength, dynamic balance control and sensory integration ability significantly influence obstacle crossing performance. We suggest an emphasis on muscle strengthening exercises (especially ankle dorsiflexors), balance training (especially forward DC), and sensory integration training to improve obstacle crossing performance in patients with PD.

## Introduction

Parkinson's disease (PD) is a neurological degenerative disease with symptoms of rigidity, tremor, bradykinesia, and impaired balance. These symptoms may lead to decreased activities and falls [1]. It has been reported that over two-thirds of community-dwelling individuals with PD experience falls once per year [2], with tripping over obstacles as the major cause. Studies have suggested that PD patients adopt different strategies to cross obstacles compared with age-matched elderly individuals, demonstrating shorter step length, larger step width, reduced stride velocity, increased double limb support time, and decreased postural stability [3]–[5]. Thus, understanding the factors associated with obstacle crossing behavior in PD patients may explain their crossing performance and help preventing possible fall risks.

Previous studies suggested that lower extremity muscle strength correlated with comfortable walking speed [5] and 6-minute walking distances [6] in PD patients. In addition, impaired dynamic balance control has been associated with gait disturbances in PD patients [7], [8]. Yang et al. [9] reported that center of mass (COM) displacement control while standing was significantly correlated with both walking speed and stride length in PD patients.

Impairments in sensory integration have been reported in individuals with PD [5]. Deficits in sensory integration of the visual, somatosensory, and vestibular systems may further contribute to postural instability [5], [10], [11] and influence both gait [5] and turning performance [12] in PD patients. Taking together, most studies indicated that muscle strength, balance and sensory integration ability correlated with level walking performance in PD patients. However, no study investigated the relationships between those factors and obstacle crossing performance or established the influencing factors for obstacle crossing performance in patients with PD. Therefore, the first purpose of this study was to identify the relationships between obstacle crossing performance and muscle strength, dynamic balance, and sensory integration. The second purpose was to identify which factors influence obstacle crossing performance in PD patients.

## Methods

### Participants

Participants were recruited from a medical center in Taiwan and were diagnosed with idiopathic PD by a neurologist. The diagnostic criteria were: (a) at least two of the following features were present: resting tremor, bradykinesia, rigidity and asymmetric onset; (b) at least one feature was tremor or bradykinesia. All participants met the following inclusion criteria: (a) Hoehn and Yahr stages I to III, (b) ability to walk independently without any walking aids, (c) stable medication usage, (d) with or without deep brain stimulation, and (e) a score of more than or equal to 24 on the Mini-Mental State Examination. The exclusion criteria were as follows: (a) unstable medical condition (eg, deep vein thrombosis, aspiration pneumonia or superimposed sepsis), (b) history of other neurological, cardiopulmonary or orthopaedic diseases known to interfere with participation in the study (eg, heart failure, hemi-neglect, or diabetic neuropathy), (c) past history of seizure, and (d) use of cardiac pacemaker. Seventy-five individuals were identified as potential participants for this study. Among these, forty-two participants provided written informed consent of all study procedures, which were approved by the Institutional Human Research Ethics Committee of Chang Gung Medical Foundation.

### Study Protocol

Forty-two patients participated in the study. All assessments were conducted with patients in the “on” state when they moved freely and easily without excessive rigidity or tremor.

### Assessments

#### Obstacle crossing performance

**Figure 1:**
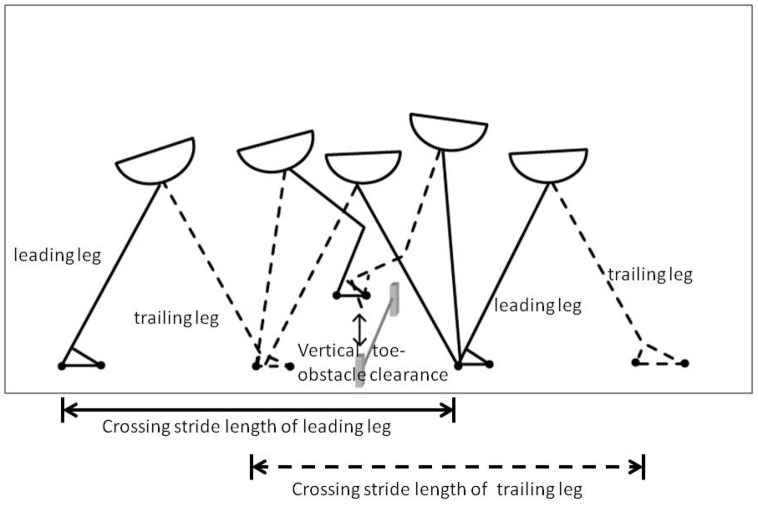
Obstacle crossing parameters.

The Liberty system (Polhtemus, Inc., Colchester, VT) was used to measure spatial temporal variables during obstacle crossing. It is an electromagnetic motion capture device for tracking three-dimensional movement at a speed of 240 updates per second. Two sensors were attached to the top of each second toe. The validity and reliability of the Liberty system have been previously established [13]. The obstacle crossing task required participants to walk on a 10-meter walkway with an obstacle positioned in the middle of the walkway. The obstacle was a plastic crossbar (60 cm long and 1.5 cm in diameter) supported by two vertical posts. The height of the obstacle was 20% of subject's leg length (14–20 cm) to emulate the height of a curb or stair. During obstacle crossing trials, participants initiated walking, stepped over the obstacle, and continued to walk to the end of the walkway at a comfortable speed. The leg that first crossed the obstacle was defined as the leading leg and the following leg as the trailing leg. Four obstacle-crossing variables are shown in Figure 1 and were analyzed as follows:

1. Crossing stride length: the distance from the heel-strike of the leading/trailing leg before the obstacle to the heel-strike of the leading/trailing leg after crossing the obstacle
2. Crossing stride velocity: the velocity from the heel-strike of the leading/trailing leg before the obstacle to the heel-strike of the leading/trailing leg after clearing the obstacle
3. Step width: the horizontal distance between the legs perpendicular to the direction of progression
4. Vertical toe-obstacle clearance: the vertical distance between the toe sensor of the leading/trailing leg and the obstacle when the toe of the leading/trailing leg was directly above the obstacle

#### Dynamic balance performance

Dynamic balance performance was assessed by the Balance Master system (NeuroCom International, Inc, USA). Limit of stability (LOS) testing was used to document dynamic balance performance [14], [15]. To assess the LOS, the subject stood on the forceplate and shifted his/her center of gravity (COG) to reach a maximal distance in the target direction as quickly and accurately as possible without moving the feet. The directions assessed included forward, right, and left. Data from the right and left directions were averaged to indicate sideward control. Movement velocity (MV), maximum excursion (ME), and directional control (DC) were collected during the LOS test in this study. MV is defined as the average speed in degrees/second in a specific direction. ME is defined as the furthest distance traveled by the COG during the trial. DC is defined as the amount of movement in the intended direction minus the amount of extraneous movement. A DC score of 100% indicates that the participant does not deviate from a straight path during the test.

#### Sensory integration ability

The sensory organization test (SOT) was assessed by the Balance Master system to represent sensory integration ability. The equilibrium score of the SOT was obtained under each of six sensory conditions. Through sway of the visual surround and support surfaces, inaccurate information is delivered to the somatosensory, visual, and vestibular systems. Somatosensory (SOM), visual (VIS) and vestibular (VEST) ratio represent the patient's ability to use inputs from the respective system to maintain balance. The sum of SOT scores is the weighted average of scores from all sensory conditions to indicate sensory integration ability [16].

#### Muscle strength

The maximal isometric muscle strength was evaluated using a handheld dynamometer (PowerTrack II; JTech Medical, USA) [17]. The muscle groups measured included hip flexors, hip extensors, knee flexors, knee extensors, ankle dorsiflexors, and ankle plantarflexors.

The testing position for the hip flexors and knee extensors was sitting with the hips and knees flexed at 90°. The testing position for the hip extensors and knee flexors was the prone position with the knee flexed at 90°. The testing position for the ankle dorsiflexors was the supine position with the hip and knee straight, and the supine position with the hip and knee flexed at 90° supported by a wooden block for the ankle plantarflexors. Subjects exerted a maximum force for 5 seconds. Three trials were obtained with a 15-second rest between trials. The average value of three trials was used for data analysis. The intra-rater reliabilities of the lower extremity muscles in normal subjects were good, with intraclass correlation coefficients ranging from 0.972 to 0.991.

#### 39-Question Parkinson's Disease Questionnaire (PDQ39)

The questionnaire was developed for PD patients to represent quality of life [18]. It contains eight dimensions with 39 items including mobility, activities of daily living, emotions, stigmas, social, cognition, communication, and body pain. Subjects filled out the questionnaire according to how often they experienced a problem during the last month. The validity of the PDQ39 was reported in a previous study [19]. A higher score represents a poor quality of life. The Chinese version of the PDQ39 was used in this study [20].

#### Falls Efficacy Scale-International (FES-I)

The FES-I is widely used in elderly persons to indicate concerns about falling. There are 16 items of functional tasks and social-related activities, scoring from 1 to 4. Subjects rated the items according to their concern about falling. A higher score indicates a greater concern about falling. The validity of the FES-I was reported in older adults [21], [22].

### Statistical Analysis

The Statistical Package for the Social Sciences 17.0 was used for statistical analysis. Descriptive statistics were gathered on means, standard deviations, and frequencies for clinical characteristics. The Pearson correlation coefficient was used to examine correlations between obstacle crossing performance and muscle strength, dynamic balance, and sensory integration ability. Those variables that significantly correlated with crossing performance were put into the regression model as independent variables. Stepwise regression analysis, performed by the forward stepwise method and controlled by disease severity, was then used to identify the most important factors influencing obstacle crossing performance. The relationships between obstacle crossing performance and the PDQ39 and FES-I were also analyzed by Pearson correlation coefficients. Significance level was set at less than 0.05.

## Results

Forty-two subjects (male: 26; female: 16) participated in the study. The mean age was 65.1±6.4 years (range: 50–84 years) with a Hoehn and Yahr stage of 1.8±0.5 (range: 1–3) and disease duration of 6.3±2.2 years (range: 1–15 years).

The relationships between obstacle crossing variables and lower extremity muscle strength are presented in Table 1. Results showed that leading leg crossing stride length correlated with muscle strength of the hip flexors, hip extensors, knee flexors, and ankle dorsiflexors. Trailing leg crossing stride length correlated with muscle strength of the hip flexors, hip extensors, knee flexors, knee extensors, and ankle dorsiflexors. Leading leg crossing velocity correlated with muscle strength of the hip flexors, hip extensors, knee flexors, knee extensors, and ankle dorsiflexors. Trailing leg crossing velocity correlated with muscle strength of the hip flexors, hip extensors, knee flexors, knee extensors, and ankle dorsiflexors.

**Table 1:** Correlations (r values) between crossing variables and lower extremity muscle strength (n = 42).

Crossing Variables	Hip		Knee		Ankle	
	flexors	extensors	flexors	extensors	dorsiflexors	plantarflexors
Crossing stride length						
Leading leg	.282*	.397*	.418**	.202	.426**	.090
Trailing leg	.326*	.457**	.425**	.307*	.440**	.081
Crossing stride velocity						
Leading leg	.365*	.486**	.496**	.337*	.543**	.146
Trailing leg	.398*	.523**	.509**	.409*	.543**	.164
Crossing step width						
Leading leg	−.084	−.222	−.22 1	−.083	−.238	−.288
Trailing leg	−.059	−.093	−.159	−.044	−.226	−.038
Vertical toe-obstacle clearance						
Leading leg	−.015	−.192	−.059	−.100	−.143	−.135
Trailing leg	−.110	−.085	−.010	−.151	−.133	−.124

*P*<0.05; **P<0.01.

The relationships between obstacle crossing variables and dynamic balance are presented in Table 2. Leading leg crossing stride length correlated with forward DC, sideward DC, and forward ME. Trailing leg crossing stride length correlated with sideward MV, forward DC, sideward DC, and forward ME. Leading leg crossing stride velocity correlated with forward DC, sideward DC, and forward ME. Trailing leg crossing stride velocity correlated with forward DC, sideward DC, and forward ME. Leading leg crossing step width correlated negatively only with sideward ME.

**Table 2:** Correlations (r values) between crossing variables and dynamic balance variables (n = 42).

Crossing Variables	Dynamic Balance in Limits of Stability Test					
	MV		DC		ME	
	forward	sideward	forward	sideward	forward	sideward
Crossing stride length						
Leading leg	.225	.329	.452**	.312*	.302*	.075
Trailing leg	.275	.344*	.486**	.400**	.404*	.239
Crossing stride velocity						
Leading leg	.241	.296	.399*	.326*	.371*	.197
Trailing leg	.260	.317	.411*	.379*	.395*	.277
Crossing step width						
Leading leg	−.037	−.051	−.031	−.106	−.132	−.432**
Trailing leg	.017	−.109	−.142	−.157	−.067	−.257
Vertical toe-obstacle clearance						
Leading leg	.164	.227	−.274	−.342*	−.080	−.151
Trailing leg	.107	.171	−.102	−.119	.015	−.021

*P*<0.05; **P<0.01. MV: movement velocity; DC: directional control; ME: maximal excursion.

The relationships between obstacle crossing variables and sensory integration ability are presented in Table 3. Leading leg crossing stride length correlated with VIS ratio, VEST ratio, and sum of SOT scores. Trailing leg crossing stride length correlated with VIS ratio, VEST ratio, and sum of SOT scores. Leading leg crossing stride velocity correlated with VIS ratio, VEST ratio, and sum of SOT scores. Trailing leg crossing stride velocity correlated with VIS ratio, VEST ratio, and sum of SOT scores. Vertical toe-obstacle clearance of the trailing leg correlated negatively with the sum of SOT scores.

**Table 3:** Correlations (r values) between crossing variables and sensory integration ability (n = 42).

Crossing Variables	Sensory Ratio of Sensory Organization Test (SOT)			
	SOM	VIS	VEST	Sum of SOT scores
Crossing stride length				
Leading leg	−.077	.435*	.355*	.350*
Trailing leg	−.027	.459*	.436*	.350*
Crossing stride velocity				
Leading leg	−.015	.404**	.362*	.366*
Trailing leg	−.033	.398**	.406**	.395*
Crossing step width				
Leading leg	.040	.117	.106	−.201
Trailing leg	−.100.	.080	.291	−.059
Vertical toe-obstacle clearance				
Leading leg	.066	−.044	−.038	−.237
Trailing leg	.161	−.257	−.014	−.313*

*P*<0.05; **P<0.01. SOM: somatosensory ratio; VIS: visual ratio; VEST: vestibular ratio.

The results of the regression analysis are shown in Table 4. Stepwise regression analysis revealed that the forward DC was the best determinant of crossing stride length, accounting for a 20.5% variance of the leading leg and a 23.7% variance of the trailing leg stride length. Ankle dorsiflexor strength was also a significant determinant. These two factors explained 37% of the variance of the leading leg and 41.2% of the trailing leg stride lengths.

**Table 4:** Stepwise regression analysis for obstacle crossing variables (n = 42).

Dependent Variables	Independent Variables	β	*R* ^2^	F	*P*
Crossing stride length					
Leading leg	Forward DC	.452	.205	9.773	<.003
	Ankle dorsiflexor strength	.407	.370	10.871	<.000
Trailing leg	Forward DC	.486	.237	11.779	<.001
	Ankle dorsiflexor strength	.419	.412	12.965	<.000
Crossing stride velocity					
Leading leg	Ankle dorsiflexor strength	.543	.294	15.861	<.000
	Forward DC	.376	.436	14.297	<.000
Trailing leg	Ankle dorsiflexor strength	.543	.295	15.863	<.000
	Forward DC	.388	.445	14.817	<.000
Crossing step width					
Leading leg	Sideward ME	−.441	.194	9.158	<.004
Trailing leg	-				
Vertical toe-obstacle clearance					
Leading leg	Sideward DC	−.342	.117	5.020	<.031
Trailing leg	SOT score	−.313	.098	4.113	<.05

For crossing stride velocity, ankle dorsiflexor strength was the best determinant, accounting for 29.4% of the variance of the leading leg and 29.5% of the variance of the trailing leg. Forward DC was also a significant determinant. These two factors explained 43.6% of the variance of the leading leg and 44.5% of the trailing leg stride velocities.

For crossing step width of the leading leg, sideward ME was the best determinant, accounting for 19.4% of the variance. However, none of our measured variables determined the variance of crossing step width of the trailing leg. For toe clearance of the leading leg, sideward DC was the best determinant, accounting for 11.7% of the variance. For toe clearance of the trailing leg, sensory integration ability was the best determinant, accounting for 9.8% of the variance.

The relationships between obstacle crossing variables and the PDQ39 and FES-I are presented in Table 5. Both crossing stride length and stride velocity correlated negatively with PDQ39 and FES-I.

**Table 5:** Correlations (r values) between crossing variables and PDQ39 and FES-I (n = 42).

Crossing Variables	PDQ39	FES-I
Crossing stride length_		
Leading leg	−.418*	−.476*
Trailing leg	−.484*	−.489*
Crossing stride velocity_		
Leading leg	−.493*	−.450*
Trailing leg	−.457*	−.484*
Crossing step width		
Leading leg	.262	.288
Trailing leg	.099	.077
Vertical toe-obstacle clearance		
Leading leg	.231	.101
Trailing leg	−.255	−.350

P<0.05.

## Discussion

Our results showed that obstacle crossing performance is influenced by lower extremity muscle strength, dynamic balance control and sensory integration ability in PD patients. Among these variables, forward DC and dorsiflexor strength were major determinants for crossing stride length and stride velocity.

In PD patients, the contribution of lower extremity strength to level walking velocity and distance were previously noted [5], [6]. From the present study, we showed a significant influence of lower extremity strength on obstacle crossing performance. Compared to level walking, the standing leg during crossing task needs additional support time for the crossing leg to produce a sufficient flexor angle to cross the obstacle. Therefore, stronger lower extremities result in larger crossing stride lengths and faster crossing velocities in PD patients. Lower extremity muscle strength also influences crossing ability in community elderly persons [23], [24].

According to our regression analysis, ankle dorsiflexor strength was the primary determining factor for crossing stride length and velocity, accounting for almost 30% of the variance. During obstacle crossing, ankle dorsiflexors contract at a sufficient flexion angle to prevent tripping. Bradykinesia and weakness are more obvious in distal muscles than in proximal muscles in PD patients [25]. Therefore, inadequate dorsiflexion control due to weakness may lead to inadequate crossing stride length and velocity. We suggest that a lower extremity muscle strengthening program should be emphasized to improve obstacle crossing performance in PD patients.

We noted that both crossing stride length and stride velocity correlated significantly with forward ME and DC. This finding infers that the better coordination and weight shifting ability while standing, the better performance of obstacle crossing. The same relationships between weight shifting ability and gait performance during level walking were noted in PD patients [8], [9].

Compared to age-matched controls from the Balance Master databank, our subjects showed significantly reduced forward MV, sideward MV, forward ME, and forward DC. Reduced balance performance may reflect bradykinesia, coordination deficits, or a fear of falls in PD patients [26].

As indicated by our regression analysis, forward DC was the determining factor for crossing stride length and velocity, together with ankle dorsiflexor strength, accounting for 37% to 44.5% of the variance. Obstacle crossing is a balance challenging task, because subjects need to move their COM forward and away from the supporting leg. A previous study noted that patients with PD crossed the obstacle with their COM closer to their supporting leg and a reduced forward movement [4]. Compared to age-matched subjects, our participants showed a deficit in forward control. As a result, they may adopt a more conservative crossing strategy, such as reduced crossing stride length and stride velocity, to minimize forward weight shifting for safety.

Our data indicated that sideway balance control correlated negatively with toe clearance and step width during crossing. From the regression model, the leading leg step width and toe-obstacle clearance were only determined by sideward ME and sideward DC, respectively. Sideward ME and DC are indicators of balance control ability. Patients with poor sideward weight shifting ability may adopt a crossing strategy that increases both toe clearance (over-lifting strategy) and step width to prevent tripping and postural instability. Previous studies also noted that patients with PD had difficulty in lengthening their step over the obstacle but increased their step width as compensation [3]. Therefore, an emphasis on balance training in the anterior direction to restore stride length and velocity, or in the sideward direction to restore toe clearance and step width, are suggested for rehabilitation programs.

We noted that VIS and VEST ratios, and the sum of SOT scores correlated positively with crossing stride length and crossing velocity. The importance of visual cues on level walking has been noted in subjects with PD [27]. Vestibular impairment can also account for postural reactions [28]. In our study, patients with PD appear to rely heavily on sensory integration ability, such as vision and vestibular inputs, during the crossing task.

From our regression analysis, the sum of SOT scores was the determining factor for toe clearance of the trailing leg, accounting for 9.8% of the variance. Compared to age-matched controls from the Balance Master databank, our participants showed a decreased sum of SOT scores. Decreased sensory integration ability could impair motor control in walking and turning in patients with PD [5], [12]. While the trailing leg crosses the obstacle, those with poor sensory integration ability may adopt an over-lifting strategy to prevent tripping. We suggest that sensory integration training be incorporated into rehabilitation programs.

Crossing performance correlated negatively with PDQ39 and FES-I scores in our study, implying that patients with PD who could cross the obstacle safely and efficiently would have better quality of life and fall-preventing confidence. Therefore, obstacle crossing performance could indicate quality of life and possible fall risk in patients with PD.

Our results indicate that lower extremity muscle strength, balance control, and sensory integration ability significantly influence obstacle crossing performance. We suggest an emphasis on muscle strengthening exercises (especially ankle dorsiflexors), balance training (especially forward DC), and sensory integration training to improve obstacle crossing performance and quality of life in patients with PD.

## References

[1] Stolze H, Klebe S, Zechlin C, Baecker C, Friege L, et al (2004) Falls in frequent neurological diseases–prevalence, risk factors and aetiology. J Neurol 251: 79–84. 14999493 10.1007/s00415-004-0276-8

[2] Ashburn A, Stack E, Pickering RM, Ward CD (2001) A community-dwelling sample of people with Parkinson's disease: characteristics of fallers and non-fallers. Age Ageing 30: 47–52. 11322672 10.1093/ageing/30.1.47

[3] Galna B, Murphy AT, Morris ME (2010) Obstacle crossing in people with Parkinson's disease: foot clearance and spatiotemporal deficits. Hum Mov Sci 29: 843–852. 19962206 10.1016/j.humov.2009.09.006

[4] Stegemoller EL, Buckley TA, Pitsikoulis C, Barthelemy E, Roemmich R, et al (2012) Postural instability and gait impairment during obstacle crossing in Parkinson's disease. Arch Phys Med Rehabil 93: 703–709. 22318131 10.1016/j.apmr.2011.11.004

[5] Nallegowda M, Singh U, Handa G, Khanna M, Wadhwa S, et al (2004) Role of sensory input and muscle strength in maintenance of balance, gait, and posture in Parkinson's disease: a pilot study. Am J Phys Med Rehabil 83: 898–908. 15624568 10.1097/01.phm.0000146505.18244.43

[6] Nocera JR, Buckley T, Waddell D, Okun MS, Hass CJ (2010) Knee extensor strength, dynamic stability, and functional ambulation: are they related in Parkinson's disease? Arch Phys Med Rehabil 91: 589–595. 20382292 10.1016/j.apmr.2009.11.026 PMC3607197

[7] Hanakawa T, Katsumi Y, Fukuyama H, Honda M, Hayashi T, et al (1999) Mechanisms underlying gait disturbance in Parkinson's disease: a single photon emission computed tomography study. Brain 122 ( Pt 7): 1271–1282. 10.1093/brain/122.7.1271 10388793

[8] Blin O, Ferrandez AM, Serratrice G (1990) Quantitative analysis of gait in Parkinson patients: increased variability of stride length. J Neurol Sci 98: 91–97. 2230833 10.1016/0022-510x(90)90184-o

[9] Yang YR, Lee YY, Cheng SJ, Lin PY, Wang RY (2008) Relationships between gait and dynamic balance in early Parkinson's disease. Gait Posture 27: 611–615. 17890091 10.1016/j.gaitpost.2007.08.003

[10] Waterston JA, Hawken MB, Tanyeri S, Jantti P, Kennard C (1993) Influence of sensory manipulation on postural control in Parkinson's disease. J Neurol Neurosurg Psychiatry 56: 1276–1281. 8270927 10.1136/jnnp.56.12.1276 PMC1015374

[11] Vaugoyeau M, Viel S, Assaiante C, Amblard B, Azulay JP (2007) Impaired vertical postural control and proprioceptive integration deficits in Parkinson's disease. Neuroscience 146: 852–863. 17367947 10.1016/j.neuroscience.2007.01.052

[12] Earhart GM, Stevens ES, Perlmutter JS, Hong M (2007) Perception of active and passive turning in Parkinson disease. Neurorehabil Neural Repair 21: 116–122. 17312086 10.1177/1545968306290674

[13] Bohannon RW, Harrison S, Kinsella-Shaw J (2009) Reliability and validity of pendulum test measures of spasticity obtained with the Polhemus tracking system from patients with chronic stroke. J Neuroeng Rehabil 6: 30. 19642989 10.1186/1743-0003-6-30 PMC2724410

[14] Liston RA, Brouwer BJ (1996) Reliability and validity of measures obtained from stroke patients using the Balance Master. Arch Phys Med Rehabil 77: 425–430. 8629916 10.1016/s0003-9993(96)90028-3

[15] Pickerill ML, Harter RA (2011) Validity and reliability of limits-of-stability testing: a comparison of 2 postural stability evaluation devices. J Athl Train 46: 600–606. 22488184 10.4085/1062-6050-46.6.600 PMC3418936

[16] Oliveira CB, Medeiros IR, Greters MG, Frota NA, Lucato LT, et al (2011) Abnormal sensory integration affects balance control in hemiparetic patients within the first year after stroke. Clinics (Sao Paulo) 66: 2043–2048. 22189728 10.1590/S1807-59322011001200008 PMC3226598

[17] Bohannon RW (1986) Test-retest reliability of hand-held dynamometry during a single session of strength assessment. Phys Ther 66: 206–209. 3945674 10.1093/ptj/66.2.206

[18] Hagell P, Nygren C (2007) The 39 item Parkinson's disease questionnaire (PDQ-39) revisited: implications for evidence based medicine. J Neurol Neurosurg Psychiatry 78: 1191–1198. 17442762 10.1136/jnnp.2006.111161 PMC2117601

[19] Jenkinson C, Fitzpatrick R, Peto V, Greenhall R, Hyman N (1997) The Parkinson's Disease Questionnaire (PDQ-39): development and validation of a Parkinson's disease summary index score. Age Ageing 26: 353–357. 9351479 10.1093/ageing/26.5.353

[20] Zhang JL, Chan P (2012) Reliability and validity of PDQ-39: a quality-of-life measure for patients with PD in China. Qual Life Res 21: 1217–1221. 21983714 10.1007/s11136-011-0026-1

[21] Helbostad JL, Taraldsen K, Granbo R, Yardley L, Todd CJ, et al (2010) Validation of the Falls Efficacy Scale-International in fall-prone older persons. Age Ageing 39: 259. 10.1093/ageing/afp224 20031925

[22] Kempen GI, Todd CJ, Van Haastregt JC, Zijlstra GA, Beyer N, et al (2007) Cross-cultural validation of the Falls Efficacy Scale International (FES-I) in older people: results from Germany, the Netherlands and the UK were satisfactory. Disabil Rehabil 29: 155–162. 17364765 10.1080/09638280600747637

[23] Lamoureux E, Sparrow WA, Murphy A, Newton RU (2003) The effects of improved strength on obstacle negotiation in community-living older adults. Gait Posture 17: 273–283. 12770641 10.1016/s0966-6362(02)00101-7

[24] Lamoureux EL, Sparrow WA, Murphy A, Newton RU (2002) The relationship between lower body strength and obstructed gait in community-dwelling older adults. J Am Geriatr Soc 50: 468–473. 11943042 10.1046/j.1532-5415.2002.50112.x

[25] Kim JW, Kwon Y, Kim YM, Chung HY, Eom GM, et al (2012) Analysis of lower limb bradykinesia in Parkinson's disease patients. Geriatr Gerontol Int 12: 257–264. 21999184 10.1111/j.1447-0594.2011.00761.x

[26] Brauer SG, Burns YR, Galley P (2000) A prospective study of laboratory and clinical measures of postural stability to predict community-dwelling fallers. J Gerontol A Biol Sci Med Sci 55: M469–476. 10952371 10.1093/gerona/55.8.m469

[27] Azulay JP, Mesure S, Blin O (2006) Influence of visual cues on gait in Parkinson's disease: contribution to attention or sensory dependence? J Neurol Sci 248: 192–195. 16765379 10.1016/j.jns.2006.05.008

[28] Vitale C, Marcelli V, Furia T, Santangelo G, Cozzolino A, et al (2011) Vestibular impairment and adaptive postural imbalance in parkinsonian patients with lateral trunk flexion. Mov Disord 26: 1458–1463. 21465552 10.1002/mds.23657

